# Gastrogastric Intussusception: A Rare Complication of Hiatal Hernias

**DOI:** 10.7759/cureus.96044

**Published:** 2025-11-03

**Authors:** Karen Lo, Nicholas Cheung, Rhett Harmon, Christine Dang

**Affiliations:** 1 Internal Medicine, Olive View-University of California Los Angeles Medical Center, Sylmar, USA

**Keywords:** abdominal pain, endoscopic reduction, gastrogastric intussusception, hiatal hernia, vomiting

## Abstract

Gastrogastric intussusception (GGI) is a rare and potentially serious condition in adults that presents with symptoms of acute abdominal pain, nausea, and vomiting. We report a case of GGI in a 58-year-old woman presenting with sudden-onset lower abdominal pain, nausea, vomiting, and constipation. She underwent a diagnostic and therapeutic endoscopic examination and was found to have a type I hiatal hernia complicated by intussusception, which was successfully reduced via insufflation and gentle pressure. Further evaluation revealed a hiatal hernia as the potential lead point, a rare occurrence in cases of adult intussusception.

## Introduction

A hiatal hernia is a condition that occurs when elements from the abdominal cavity herniate through the diaphragmatic hiatus into the chest cavity. It is a relatively common condition seen in approximately 55%-60% of individuals over the age of 50, although only about 9% of cases manifest symptoms [1]. Most typically, gastroesophageal reflux disease is the presenting complaint, with symptoms of heartburn, regurgitation, reflux, or dysphagia. Diagnosis may be done via radiography, endoscopy, or manometry. The etiology of hiatal hernias has been implicated with a variety of factors, ranging from trauma, congenital malformations, increased body mass index, and age to iatrogenic factors such as past gastroesophageal surgeries or other procedures [2].

The Skinner and Belsey classification divides hiatal hernias into four types based on their anatomical positioning: type I (sliding hernia with displacement of the gastroesophageal junction (GEJ)), type II (paraesophageal, with movement of the stomach into the mediastinum parallel to the esophagus while the GEJ remains in its normal anatomical position), type III (mixed sliding and paraesophageal), or type IV (complex hernia with herniation of other abdominal organs). Type I hiatal hernias are by far the most common, comprising 85%-95% of all hiatal hernias [3]. These hernias are thought to result from laxity of the phrenoesophageal membrane, allowing transposition of the GEJ upward with displacement of the stomach [3].

Moreover, for those experiencing gastroesophageal reflux disease, endoscopic evaluation of the anatomical competence of the GEJ provides valuable information that can help guide subsequent management. The Hill system categorizes the gastroesophageal flap valve into one of four grades: grade I (flap valve with consistent tight closure around the endoscope), grade II (less defined flap valve with respiration-dependent, incomplete closure around the endoscope), grade III (ill-defined flap valve without closure around the endoscope), and grade IV (absent flap valve with permanent opening of the GEJ) [4,5]. Higher Hill grades are associated with increased reflux frequency and a poor response to proton pump inhibitor treatment [5].

Proper hernia classification aids in the management of symptoms. Initially, all patients (types I-IV) presenting with gastroesophageal reflux symptoms are treated with medical therapy such as proton pump inhibitors. For those with lower Hill grades I-II and small hiatal hernias (<2 cm), the prognosis is generally favorable, with a lower risk of reflux complications (i.e., ulcers, strictures, and Barrett’s mucosa); lifestyle modifications and proton pump inhibitors are typically the first-line treatment. In contrast, those with Hill grades III-IV and larger hiatal hernias (>2 cm) are at higher risk of reflux-related complications and are more likely to require escalation beyond medical therapy. The American Society for Gastrointestinal Endoscopy recommends that these patients be evaluated for hiatal hernia repair or surgical fundoplication [6]. Additionally, those with paraesophageal hernias (types II-IV) are at higher risk of complications such as intussusception or gastric volvulus, which are considered surgical emergencies. Thus, it is currently recommended that those who are asymptomatic and less than 60 years old or are symptomatic should undergo surgical repair [7,8].

Here, we present a case of a 58-year-old woman found to have a gastrogastric intussusception, where it was suspected that her hiatal hernia served as the lead point. Intussusception in adults is exceedingly rare and comprises 5% of all intussusceptions. Furthermore, only roughly 10% of adult cases involve the stomach [9,10]. We aim to showcase a rare example of a potentially life-threatening complication from hiatal hernias and discuss the underlying pathophysiology and subsequent management.

## Case presentation

**Figure 1:**
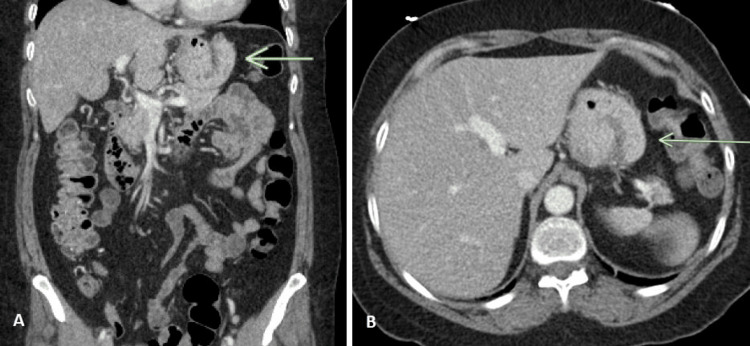
CT of the abdomen and pelvis demonstrating folding of the gastric cardia onto itself on both coronal (A) and axial (B) views (arrows) CT: computed tomography

A 58-year-old woman with a past medical history of pre-diabetes, hypertension, and cholelithiasis status-post cholecystectomy, presented to the emergency department for a one-day history of sudden-onset lower abdominal pain. She reported nausea, non-bloody vomiting, and a 10-day history of constipation without any bowel movements. The patient denied any unintentional weight loss but endorsed to providers that she had been unable to tolerate oral intake since her abdominal pain started. Initial vital signs were as follows: temperature of 37.1°C, blood pressure of 135/90, heart rate of 90 beats/minute, and respiratory rate of 22 breaths/minute with oxygen saturation of 98% on room air. Body mass index on admission was 28.09. Physical examination was significant for diffuse abdominal tenderness to palpation without rebound or guarding. Laboratory studies, including leukocytes, lipase, and lactate, were within normal limits or unchanged from baseline (Table 1). Initial abdominal X-ray showed normal stool and gas pattern without evidence of an ileus. Computed tomography (CT) of the abdomen demonstrated pathologic folding of the gastric cardia, consistent with gastric intussusception (Figure 1).

**Table 1:** Initial laboratory results upon admission AST: aspartate aminotransferase, ALT: alanine aminotransferase

Laboratory parameter	Value
White blood cell	6.7 K/cumm
Hemoglobin	11.7 g/dL
Platelets	635 K/cumm
Sodium	137 mmol/L
Potassium	4.1 mmol/L
Chloride	100 mmol/L
Bicarbonate	25 mmol/L
Anion gap	12 mmol/L
Blood urea nitrogen	10 mg/dL
Creatinine	0.64 mg/dL
Glucose	92 mg/dL
Calcium	9 mg/dL
Magnesium	2.5 mg/dL
Alkaline phosphate	216 U/L
AST	38 U/L
ALT	29 U/L
Total bilirubin	0.3 mg/dL
Direct bilirubin	0.1 mg/dL
Lipase	39 U/L
Lactate	1.6 mmol/L
Troponin	<0.010 ng/mL

**Figure 2:**
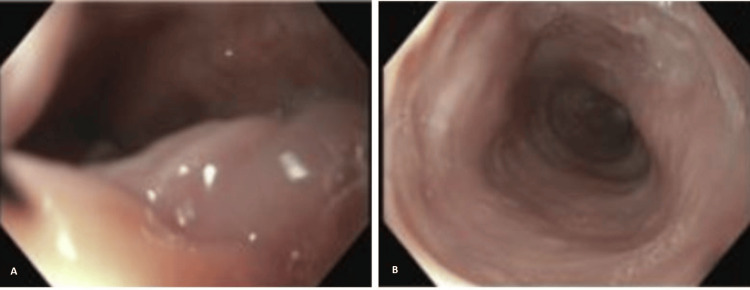
Endoscopy revealing prominent gastric folds abutting the distal esophagus (A) with subsequent opening after insufflation and gentle guidance with gastroscope (B)

Gastroenterology was consulted for emergent endoscopy, during which prominent gastric folds were visualized abutting the distal esophagus at the gastroesophageal junction but were reducible with insufflation and gentle pressure (Figure 2). Subsequent inspection of the gastric mucosa showed no evidence of neoplasms. However, a Hill grade IV hiatal hernia with an entrapped gastric fold was observed, and this was suspected to be the lead point for the intussusception.

Following the procedure, the patient’s symptoms resolved, and she was able to subsequently tolerate normal oral intake with regular bowel movements. She was discharged on hospital day 2 with plans for repeat upper gastrointestinal studies to be done to further investigate the utility of hiatal hernia reduction and possible fundoplication due to the high risk of recurrence of her intussusception.

## Discussion

Intussusception occurs when a portion of the gastrointestinal tract telescopes into itself. This can cause non-specific symptoms, including fever, abdominal pain, constipation, and nausea. The time course of symptoms can vary, with past reports of both acute and chronic symptoms as part of the disease [8,9]. Prompt recognition is crucial as delayed treatment can lead to serious complications such as bowel obstruction and ischemia. This is especially important in adults, as intussusception is exceedingly less common compared to the pediatric population and only comprises roughly 5% of all intussusception cases [10].

Traditionally, intussusception in adults has been associated with polypoidal lead points, including neoplasms, lipomas, and hyperplastic polyps, as opposed to cases in children, where approximately 90% are idiopathic [11,12]. Additionally, most cases of intussusception in adults have been reported in the small bowel, with only 10% of cases reported in the stomach or at the site of a surgical stoma [10,11,13].

Workup on intussusception should include a thorough physical examination, which often reveals a palpable abdominal mass most often located in the right upper quadrant or epigastric area due to the telescoped bowel segments. Abdominal tenderness can be generalized or localized, but in advanced cases, signs of peritonitis, including guarding, rigidity, and rebound tenderness, can be appreciated. Lastly, the rectal examination may reveal blood mixed with mucus in response to mechanical and ischemic stress on the bowel wall [14]. Initial imaging studies include an abdominal ultrasound, which may detect the invaginated portion of the bowel, which appears as rings on a target. Ultrasound has a high sensitivity (97.9%) and specificity (97.8%) compared to abdominal radiographs (sensitivity: 62.3%, specificity: 86.7%) [15]. Other imaging modalities include contrast enema, which can be both diagnostic and therapeutic by reducing telescoped bowel utilizing hydrostatic and pneumatic pressure [16].

In our patient’s case, the site of intussusception was at the gastroesophageal junction, without an associated polypoidal lead point. The gastroesophageal junction is an exceedingly rare site of intussusception, with very few documented cases. In one prospective series, severe vomiting or retching was identified to be a risk factor for patients to develop gastroesophageal intussusception, with the potential for development of Mallory-Weiss tears or esophageal perforation [17]. Another case report describing a 68-year-old man with gastrogastric intussusception and no polypoidal lead point posits that a combination of anatomical distortions and an elevated intra-abdominal pressure from ascites, hiatal hernia, and portal hypertension is an alternative mechanism for the development of intussusception [18]. In a similar fashion for our patient, we posit that with the presence of a Hill grade IV gastroesophageal junction, there are likely inherent laxities and abdominal distortions of the surrounding gastric ligaments that can serve as traction points. Combined with her history of subacute nausea and vomiting, the resulting increased intra-abdominal pressure contributed to the development of her intussusception.

Due to the rarity of adult intussusception, there are currently no clear universal guidelines for treatment in adults compared to the pediatric population. In children, initial treatment steps in hemodynamically stable patients without clinical symptoms of peritonitis involve non-surgical options. These include pneumatic and hydrostatic enemas, with a systematic review by the American Pediatric Surgical Association Outcomes reporting pneumatic reduction to be successful in approximately 82.7% of cases, while hydrostatic reduction has a success rate of 69.6% [17]. Surgical interventions such as laparoscopic or open reduction are indicated if there are signs of hemodynamic instability, bowel necrosis, perforation, or failures of non-operative reductions [17]. Conversely, guidelines for adult intussusception are not as clear. It had previously been considered an indication for surgery, given the high rates of malignancy and other structural abnormalities that may act as lead points [19]. However, with the increasing use of CT imaging, there has been an increased recognition of transient intussusception in adults, many of which were idiopathic; in several case studies, these patients had been successively treated with conservative management (intravenous fluids, bowel rest, and serial abdominal examinations) [19-21]. It has thus been suggested that intussusception cases with a length of <3.5 cm are likely to be self-limiting, and it would be reasonable to opt for conservative management if there is low suspicion for gut ischemia, necrosis, and malignancy [21]. Furthermore, there is unfortunately not a lot of data regarding the risk of recurrence or long-term outcomes. One retrospective study noted 10 cases of recurrent intussusception out of a total of 148 patients, with a median time to intussusception being 64 days; however, they found no relationship between demographic factors, presenting symptoms, management, and recurrence [22]. Therefore, close long-term follow-up after initial diagnosis is essential to monitor for any recurrence of intussusception and its related complications.

Prompt diagnosis and treatment of intussusception is important to prevent potentially life-threatening complications, such as bowel ischemia, obstruction, and perforation. Fortunately, our patient received timely intervention, and this case was a rare example of a non-malignant anatomical etiology that can predispose patients to upper gastrointestinal intussusception.

## Conclusions

Gastrogastric intussusception due to a hiatal hernia, although rare, should be considered in the differential diagnosis of patients with a history of hiatal hernia, presenting with acute abdominal pain and associated gastrointestinal symptoms. Timely imaging and endoscopic intervention can lead to favorable outcomes and prevent complications such as necrosis, perforation, or sepsis. Further workup should also be done to exclude predisposing risk factors such as the existence of a lead point lesion or past gastric surgery or manipulation; however, as is demonstrated in this case, such risk factors are not necessary for intussusception to occur.
